# Cortisol reactivity to psychosocial stress in vulnerable and grandiose narcissists: An exploratory study

**DOI:** 10.3389/fpsyg.2022.1067456

**Published:** 2023-01-06

**Authors:** Javier I. Borráz-León, Alena Spreitzer, Coltan Scrivner, Mitchell Landers, Royce Lee, Dario Maestripieri

**Affiliations:** ^1^Institute for Mind and Biology, The University of Chicago, Chicago, IL, United States; ^2^Department of Comparative Human Development, The University of Chicago, Chicago, IL, United States; ^3^Department of Psychology, The University of Chicago, Chicago, IL, United States; ^4^Department of Psychiatry and Behavioral Neuroscience, The University of Chicago, Chicago, IL, United States

**Keywords:** vulnerable narcissism, grandiose narcissism, mixed-type narcissism, cortisol reactivity, psychosocial stress, trier social stress test

## Abstract

**Introduction:**

Narcissistic personality manifests itself in at least two different forms: grandiose and vulnerable. In the present study, we compared cortisol and emotional responses to psychosocial stress between subjects high in vulnerable and grandiose narcissism scores, and examined possible associations between narcissism, other personality traits, and stress responses. We hypothesized that subjects with higher scores of vulnerable narcissism would show stronger emotional and physiological reactivity than those with high scores of grandiose narcissism.

**Methods:**

A final sample of forty-seven participants underwent a Trier Social Stress Test (TSST), provided saliva samples to assess cortisol levels, and completed several personality questionnaires.

**Results:**

Consistent with our hypothesis, subjects with higher scores of vulnerable narcissism had a stronger cortisol and emotional response than those with high scores of grandiose narcissism. Vulnerable narcissism was positively correlated with schizotypal traits, while grandiose narcissism was positively correlated with psychopathic traits. Participants with a mixed-type of narcissism were also discussed.

**Discussion:**

This study provides the first evidence of differential physiological and emotional reactivity to social evaluation threat according to scores of vulnerable and grandiose narcissism. Since this is an exploratory study, the results must be interpreted with caution. However, the results will be informative for future confirmatory research with larger and more heterogeneous samples.

## 1. Introduction

Personality disorders probably represent the extreme manifestation of traits that are normally distributed in the population. They have genetic and environmental components and may be adaptive or maladaptive depending on the circumstances (Del Giudice, 2018; Hunt and Jaeggi, 2022). The DSM-5 description of Narcissistic Personality Disorder (NPD) emphasizes a chronic pattern of dysfunction in relationships and trait-like personality features of arrogance, grandiosity, and lack of empathy (Gore and Widiger, 2016). The DSM-5 contains both a categorical (NPD) and dimensional (narcissistic traits) representation of narcissism. Non-pathological narcissistic traits are defined as “a relatively stable individual difference consisting of grandiosity, self-love and inflated self-views” (Campbell et al., 2011). In both NPD and non-pathological narcissism, self-identity and self-image are thought to depend on constant external validation from other individuals (e.g., their praise and adulation) or on continuous positive feedback through accomplishment and success (Campbell et al., 2006; Jones and Paulhus, 2011; Pincus, 2013; Roche et al., 2013). In this view, the outward confidence shown by subjects with strong narcissistic traits may be a self-regulation strategy to cope with low self-esteem (Bosson et al., 2003). The role of self-esteem in narcissism is a contentious issue in the literature, as narcissistic traits such as grandiosity and assertiveness may be displayed to conceal deep-seated feelings of inferiority, as claimed by the mask model (Miller et al., 2017); reports on the association between self-esteem and narcissistic traits range from negative to positive, complicating a possible association between self-esteem and narcissism subtypes (see Miller et al., 2017 for a review). There is evidence suggesting that narcissism may also be associated with schizotypy, psychopathy (March and Springer, 2019), and autistic-like traits (Cheek and Norem, 2022), as well as non-pathological personality traits such as extraversion, neuroticism, and agreeableness (Campbell and Crist, 2020; Zajenkowski and Szymaniak, 2021).

Acknowledging the divergent and sometimes contradictory findings obtained with individuals with NPD or non-pathological narcissistic traits, the field recognizes at least two subtypes of narcissism: vulnerable and grandiose (Wink, 1991; Jauk et al., 2017; Coleman et al., 2019; Jauk and Kanske, 2021). Individuals who express traits associated with vulnerable narcissism are more likely to be introverted, usually exhibit an insecure/fearful attachment style, and tend to show traits associated with negative emotionality such as anxiety, depression, and hostility, whereas individuals who express traits associated with grandiose narcissism are more likely to be extraverted and be at least outwardly dismissive of attachment relationships (Dickinson and Pincus, 2003; Miller et al., 2010; Campbell and Miller, 2013; Pincus et al., 2015; Miller et al., 2017). Negative associations between grandiose narcissism and some indicators of negative emotionality such as depression and sadness have also been reported (*cf*. Czarna et al., 2018). In addition to cognitive differences related to self-appraisal and interpretation of other individuals and their behavior (Pincus et al., 2009; Zajenkowski et al., 2018), differences in emotion regulation have been hypothesized to separate individuals with vulnerable and grandiose narcissistic traits, especially when their ego is threatened. For example, subjects who express traits associated with vulnerable narcissism appear to be more prone to anxiety, stress, and fear, whereas subjects who express traits associated with grandiose narcissism are more prone to anger and are more resilient to stress (e.g., Besser and Priel, 2010; Roche et al., 2013; Ng et al., 2014; Fernie et al., 2016; Sękowski et al., 2021; Underwood et al., 2021). As a notable example, Besser and Priel (2010) compared emotional reactions to two threat conditions (i.e., achievement failure and interpersonal rejection) between individuals high in grandiose narcissism and individuals high in vulnerable narcissism. The authors found that under a high-level interpersonal threat, but not a high-level achievement-threat, high scores of vulnerable narcissism were associated with greater change in negative outcomes, whereas under a high-level achievement-threat, but not a high-level interpersonal threat, high scores of grandiose narcissism predicted greater change in negative outcomes, supporting the claim of differential emotional responses under different contexts between subjects high in vulnerable and grandiose narcissism scores.

Given the possibility that outward behavior may not be a reliable index of underlying psychobiological processes, an important question regarding the validity of the two subtypes of narcissism is whether they are separable biologically. There is some evidence indicating possible psychophysiological differences between individuals with vulnerable and grandiose narcissistic traits. For example, Kelsey et al. (2002) found that women with characteristics of grandiose narcissism have a heightened cardiac pre-rejection period (i.e., the interval between myocardial contractile force and aortic opening reflecting sympathetic control on the hearth), whereas women with characteristics of vulnerable narcissism have a diminished skin conductance response after two stress tasks involving mental arithmetic and the Thematic Apperception Test. Given the hypothesized self-regulatory role of narcissistic fluctuations of self-esteem in the face of psychosocial stress, the functioning of the hypothalamic–pituitary–adrenal (HPA) axis may also reflect such differences. Some data suggest that narcissism is a façade, masking interpersonal hypersensitivity and stress reactivity, similar to that observed in borderline personality disorder (Gunderson and Lyons-Ruth, 2008). Consistent with this hypothesis, in a study of 129 men, baseline levels of salivary cortisol were found to be associated with grandiose narcissism (as a component of the Dark Triad of personality, *cf*. Jones and Paulhus, 2011), whereas no association was found with psychopathy or Machiavellianism (Pfattheicher, 2016). In another study, Reinhard et al. (2012) reported that ‘unhealthy narcissism’ (high grandiose narcissism scores), predicted higher basal salivary cortisol in men while a tendency was found for women. However, one of the largest studies so far (*n* = 366) failed to find any relationship between narcissism and baseline cortisol levels (Wardecker et al., 2018).

It is worth emphasizing that the cortisol response to a stressful situation was not assessed in any of these previous studies. However, in three studies that examined the relationship between grandiose narcissism (but not vulnerable narcissism) and HPA axis reactivity to psychosocial stress, the results showed that (*a*) cortisol response following a public speaking task was elevated in individuals with grandiose narcissism (Edelstein et al., 2010), (*b*) that grandiose narcissism, as measured by the Narcissistic Personality Inventory (NPI), was positively correlated with increased cortisol levels in association with perceptions of negative emotions across 3 days (Cheng et al., 2013), and (*c*) that, contrary to these two previous studies, grandiose narcissism was associated with decreased cortisol release after the social stress of telling a lie (Dane et al., 2018). Given that there may be further unpublished non-significant results concerning the association between narcissism and HPA axis function and that studies on vulnerable narcissism and HPA axis function are lacking, this research is difficult to interpret. Methodological differences among the studies could also account for inconsistencies in previous findings. Thus, the hypothesis of divergent mechanisms of psychobiological stress-coping in grandiose and vulnerable narcissism remains open and is just beginning to be empirically explored.

To the best of our knowledge, no previous study has investigated possible differences in responses to the TSST between subjects with vulnerable and grandiose narcissism within the same study. The aims of this exploratory study were therefore (1) to compare emotional and cortisol responses to the TSST between individuals with vulnerable and grandiose narcissistic traits and (2) to examine possible associations between narcissism, other personality traits, and stress responses. For the first aim, we predicted that individuals with vulnerable narcissistic traits would show stronger emotional and physiological responses to the TSST than subjects with grandiose narcissistic traits. For the second aim, we predicted differences in personality correlations between individuals high in grandiose narcissism and individuals high in vulnerable narcissism.

## 2. Materials and methods

### 2.1. Participants

Participants were 56 self-reported healthy individuals (18 male, age: *M* = 27.39, *SD* = 9.89; 32 female, age: *M* = 22.72, *SD* = 3.41; age range: 18–53 years; six individuals did not provide information about their sex) recruited on the University of Chicago campus through fliers, Marketplace, and a human subject recruitment website (Sona System). The inclusion criteria for both sexes were the following: not having chronic diseases (e.g., diabetes or cancer) and not being subjected to hormonal therapy or psychiatric medication. Approximately 33% of study participants reported their race as Asian, 19% Black, 12% Hispanic/Latino, 33% White, and 2% Other. All study participants completed a written informed consent form before participating in the study. The study adheres to the Declaration of Helsinki and was approved by the Social Science Institutional Review Board at the University of Chicago.

### 2.2. Experimental procedure

Participants were asked to remotely complete several questionnaires at least 24 h before their scheduled laboratory visit. These questionnaires included a demographic information questionnaire (with questions about the participants’ age, ethnicity, sexual orientation, SES, and marital status). Since there is evidence suggesting that narcissism (both grandiose and vulnerable) is associated with traits of other personality disorders (e.g., March and Springer, 2019), the following scales were also used: the brief and revised version of the Schizotypal Personality Questionnaire (SPQ-BR; Cohen et al., 2010), the Narcissistic Personality Inventory (NPI; Raskin and Hall, 1979), the Hypersensitive Narcissism Scale (HSNS; Hendin and Cheek, 1997), the Autism Spectrum Quotient (AQ; Baron-Cohen et al., 2001), the Psychopathic Personality Questionnaire (PPI-R; Nikolova, 2009), and the HEXACO Personality Inventory-Revised (HEXACO-PI-R; Ashton and Lee, 2009). See Table 1 for further details on the scales.

**Table 1:** Overview of measures and scales used in this study.

Title	# of items	Subscales	Example item	Scale	Cronbach’s *α* in the present study
Brief and revised version of the Schizotypal personality questionnaire (SPQ-BR)	32	- Ideas of reference - Social anxiety - Odd beliefs - Unusual experiences - Eccentric behavior - No close friends - Odd speech - Constricted affect - Paranoid ideation - Cognitive perceptual - Interpersonal - Disorganized	- Do you sometimes feel that people are talking about you? (ideas of reference) - I sometimes avoid going to places where there will be many people because I will get anxious (social anxiety) - I rarely laugh and smile (constricted affect)	0 = strongly disagree 4 = strongly agree	0.91
Narcissistic personality inventory (NPI)	40 paired statements	N/A	(a) I would do almost anything on a dare or (b) I tend to be a fairly cautious person	To choose which one is closest to your feelings	0.80
Hypersensitive narcissism scale (HSNS)	10	N/A	My feelings are easily hurt by ridicule or the slighting remarks of others	1 = very uncharacteristic or untrue, strongly disagree 5 = very characteristic or true, strongly agree	0.74
Autism spectrum quotient (AQ)	50	N/A	I like to plan any activities I participate in carefully	1 = strongly disagree 4 = strongly agree	0.50
Psychopathic personality questionnaire (PPI-R)	144	- Machiavellian egocentricity - fearlessness - rebellious non-conformity - blame externalization - stress immunity - cold heartedness - social influence - carefree non-planfulness	- Sometimes I wake up feeling nervous without knowing why (stress immunity) - I would find the job of a movie stunt person exciting (fearlessness) - I find it hard to make small talk with people I do not know well (social influence)	1 = False 4 = True	0.82
HEXACO personality inventory-revised (HEXACO-PI-R)	60	- Honesty-Humility - Emotionality - Extraversion - Agreeableness - Conscientiousness - Openness to experience	- I would not use flattery to get a raise or promotion at work, even if I thought it would succeed (honesty-humility) - I plan ahead and organize things, to avoid scrambling at the last minute (conscientiousness)	1 = strongly disagree 5 = strongly agree	0.75
State–Trait anxiety inventory (STAI)	20	N/A	I worry too much over something that does not really matter	1 = almost never 4 = almost always	0.48

Participants were later asked to come in person to the lab, between 12 PM and 5 PM. Upon arrival, participants were taken to the testing room, where they provided a baseline saliva sample and completed the State–Trait Anxiety Inventory (STAI; Spielberger et al., 1999) and a questionnaire that evaluates 7 emotional states (i.e., anger, shame, happiness, sadness, fear, shame behavior, and devaluation). A single item was used to assess each one of the first five emotions, nine items to assess the sixth (Cronbach’s *α* = 0.93), and four items to assess the last one (Cronbach’s *α* = 0.68). All items were evaluated using a 7-point Likert-scale with values ranging from 1 = *not at all* to 7 = *extremely*. At the end of this period, they provided a second saliva sample and underwent a Trier Social Stress Test. A third saliva sample was collected 10–15 min after the TSST had ended. All participants completed the STAI and the questionnaire on emotional states again after the TSST, after which they were debriefed and compensated with $15.

### 2.3. Trier social stress test

The Trier Social Stress Test (TSST; Kirschbaum et al., 1993) is a standardized task used to study responses to mild psychosocial stress in a laboratory setting. Some inter-individual variation in both self-reported anxiety and cortisol measures in response to the TSST is accounted for by personality variation; for example, introverted individuals are typically more physiologically reactive than extraverted ones (e.g., Wilson et al., 2015). Since the TSST has consistently shown to induce increases in cortisol levels, it can be used as a reliable test of induced physiological stress (e.g., Kexel et al., 2021).

In the current study, the experimenter explained to each participant that he or she would be giving a 5-min impromptu presentation about himself or herself for a mock job interview. Each presentation took place in front of a “selection committee” composed of two unfamiliar confederates (“judges”) trained to maintain neutral facial expressions and to provide no emotional feedback to the participant (Kim et al., 2018). The confederates wore lab coats and pretended to write notes on their clipboard throughout the TSST. Each participant was informed that he or she must keep speaking for the entire 5 min and that the presentation was being videotaped for subsequent analyses of content and non-verbal behavior. If the participant stopped speaking before the 5 min were up, the judges waited in silence for the participant to resume or otherwise prompted the participant to continue. The judges then asked each participant to perform an arithmetic calculation (serially subtracting the number 17 from 2,023) out loud for another 5 min or until he or she reached zero. If the participant made a mistake, he or she was notified and asked to restart from 2023. After this task, the confederates thanked the participant and left the room.

### 2.4. Saliva sample collection and hormonal assays

All saliva samples were collected between 12:30 PM and 4:30 PM. Saliva was collected by passive drool into plastic tubes. Saliva samples were stored in a refrigerator at −20°F. Samples were assayed for cortisol concentrations using ELISA kits from the manufacturer (Salimetrics Inc.). The intra-assay CV based on concentration was 4.75% and the inter-assay CV based on concentration was 6.28%.

### 2.5. Data analysis

Nine participants were removed from the sample since they did not fully complete their questionnaires, or their cortisol concentrations could not be successfully quantified in their saliva samples. The statistical analyses were performed on a final sample of 47 subjects (17 male, age: *M* = 27.18, *SD* = 9.11, 30 female, age: *M* = 22.63, *SD* = 3.46). The data that were not normally distributed, were log-transformed to improve normality (e.g., see Luoto et al., 2021).

A *t*-test for independent samples was used to compare male and female participants on some variables of interest. Paired sample *t*-tests were run to measure changes in cortisol levels and emotions before and after the TSST. Since no significant statistical differences between the baseline and the pre-TSST saliva sample were observed (*t* = 0.095, *p* = 0.925), we carried out the rest of our analyses using the average value of the baseline and the pre-TSST sample as the pre-TSST cortisol level. Cortisol change was measured as the difference between post-TSST and the averaged pre-TSST cortisol levels. Partial correlations (controlling for age and sex) were used to measure relationships between cortisol levels (pre-TSST, post-TSST, and cortisol change) and the study variables.

Descriptive values of vulnerable and grandiose narcissism were as follows: *M* = 29.94, *SD* = 5.81,^1^ median = 30.00; *M* = 14.43, *SD* = 6.69,^2^ median = 13.00, respectively. Based on the broad consensus that there are at least two different subtypes of narcissism (e.g., Campbell and Crist, 2020; Kaufman et al., 2020) and as an exploratory approach to further analyze the association between narcissism, cortisol reactivity, and changes in emotion, we grouped the participants into three narcissism categories as follows: subjects with both HSNS scores above the median and NPI scores below the median were considered as subjects high in *vulnerable narcissism* (*n* = 12, 8 female), whereas subjects with both HSNS scores below the median and NPI scores above the median were considered as subjects high in *grandiose narcissism* (*n* = 7, 4 female). The rest of the participants were considered as subjects with a *mixed-type of narcissism* (*n* = 28, 18 female). The mixed-type group was mainly composed by subjects who have a mixture of vulnerable and grandiose narcissistic traits as their scores ranged from low to high in both vulnerable and grandiose narcissism (see Campbell and Crist, 2020 for a similar classification of subjects with narcissistic traits into these three groups; see also Olsen and Stekelberg, 2016; Kim and Jang, 2018 for a similar grouping technique). An ANOVA, with Bonferroni as *post-hoc* tests, was run to assess differences in cortisol changes between the three narcissism categories. The data were analyzed using SPSS version 25 (SPSS Inc., Chicago, IL, United States). All tests were two-tailed, and statistical significance was set at *p* ≤ 0.05.

## 3. Results

### 3.1. Sex differences

Men were, on average, older than the women but the difference was not statistically significant. Women scored higher than men in the schizotypal traits “disorganized” and “emotionality,” and somewhat higher in “odd speech.” Men scored higher than women in honesty-humility and the psychopathic trait “stress immunity,” and lower than women in the psychopathic trait “carefree non-planfulness.” No significant sex differences were found for the other study variables (Supplementary Table S1).

### 3.2. Cortisol, narcissism, and other personality traits

Partial correlation results can be found in Supplementary Table S2. In summary, pre-TSST cortisol was negatively associated with the schizotypal traits “ideas of reference” and “social anxiety,” and positively associated with the psychopathic trait “stress immunity.” No significant correlations between pre-TSST cortisol and other personality traits were found. No significant correlations were found between post-TSST cortisol and any of the personality measures.

**Figure 1:**
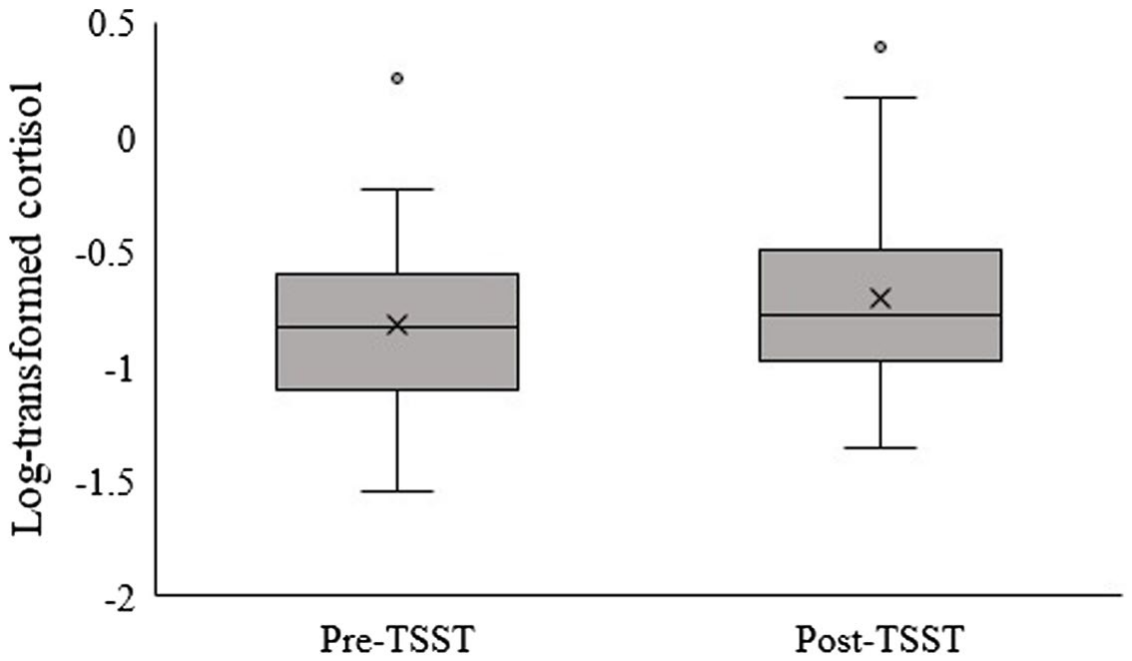
Cortisol levels (log-transformed) increased after the TSST (*t* = −3.230, *p* = 0.002, *d* = 0.48).

Cortisol levels significantly increased after the TSST (*t* = −3.230, *p* = 0.002, *d* = 0.48; Figure 1). Significant positive correlations between cortisol change and the schizotypal traits “social anxiety” and “odd speech” were found (*r* = 0.427, *p* = 0.003; *r* = 0.295, *p* = 0.049, respectively). A significant negative correlation between NPI scores and cortisol change (*r* = −0.353, *p* = 0.017) and a tendency for a positive correlation between HSNS scores and cortisol change were also found (*r* = 0.287, *p* = 0.056).

### 3.3. Narcissism subtypes

**Figure 2:**
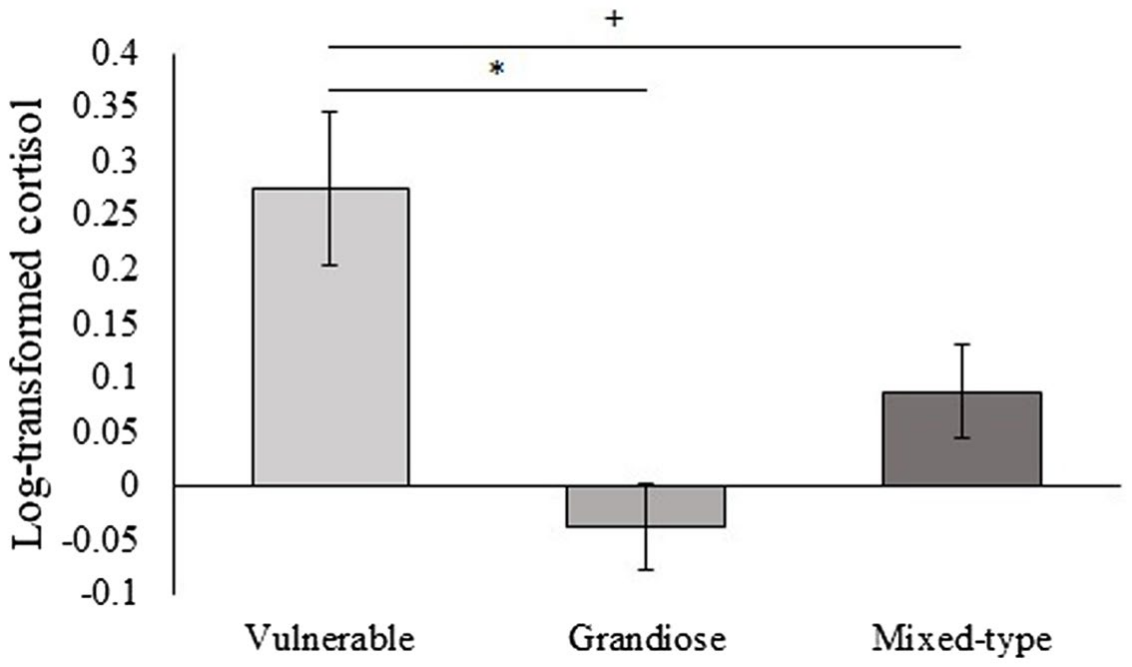
Differences in cortisol changes (log-transformed) between the three narcissism categories [*F*_(2,44)_ = 4.986, *p* = 0.011, *η*^2^ = 0.185]. Individuals high in vulnerable narcissism scores had greater cortisol response than individuals high in grandiose narcissism scores (*Bonferroni *p* = 0.015) and tend to have a greater response than individuals with mixed-type narcissism (+Bonferroni *p* = 0.056).

The analysis indicated that subjects with vulnerable narcissistic traits had a significantly greater increase in their cortisol levels following the TSST than subjects with grandiose narcissistic traits (*M* = 0.27 ± *SD* = 0.24 vs. *M* = −0.03 ± *SD* = 0.10; *p* = 0.015, respectively) and a tendency for greater increase in cortisol than subjects with a mixed-type (*M* = 0.27 ± *SD* = 0.24 vs. *M* = 0.08 ± *SD* = 0.22; *p* = 0.056, respectively; Figure 2). Since previous research has reported differences in cortisol responses to the TSST according to extraversion (e.g., Wilson et al., 2015), we also performed an ANCOVA controlling for this trait. Results remained significant after controlling for extraversion [*F*_(2,43)_ = 4.226, *p* = 0.021, *η*^2^ = 0.164].

**Figure 3:**
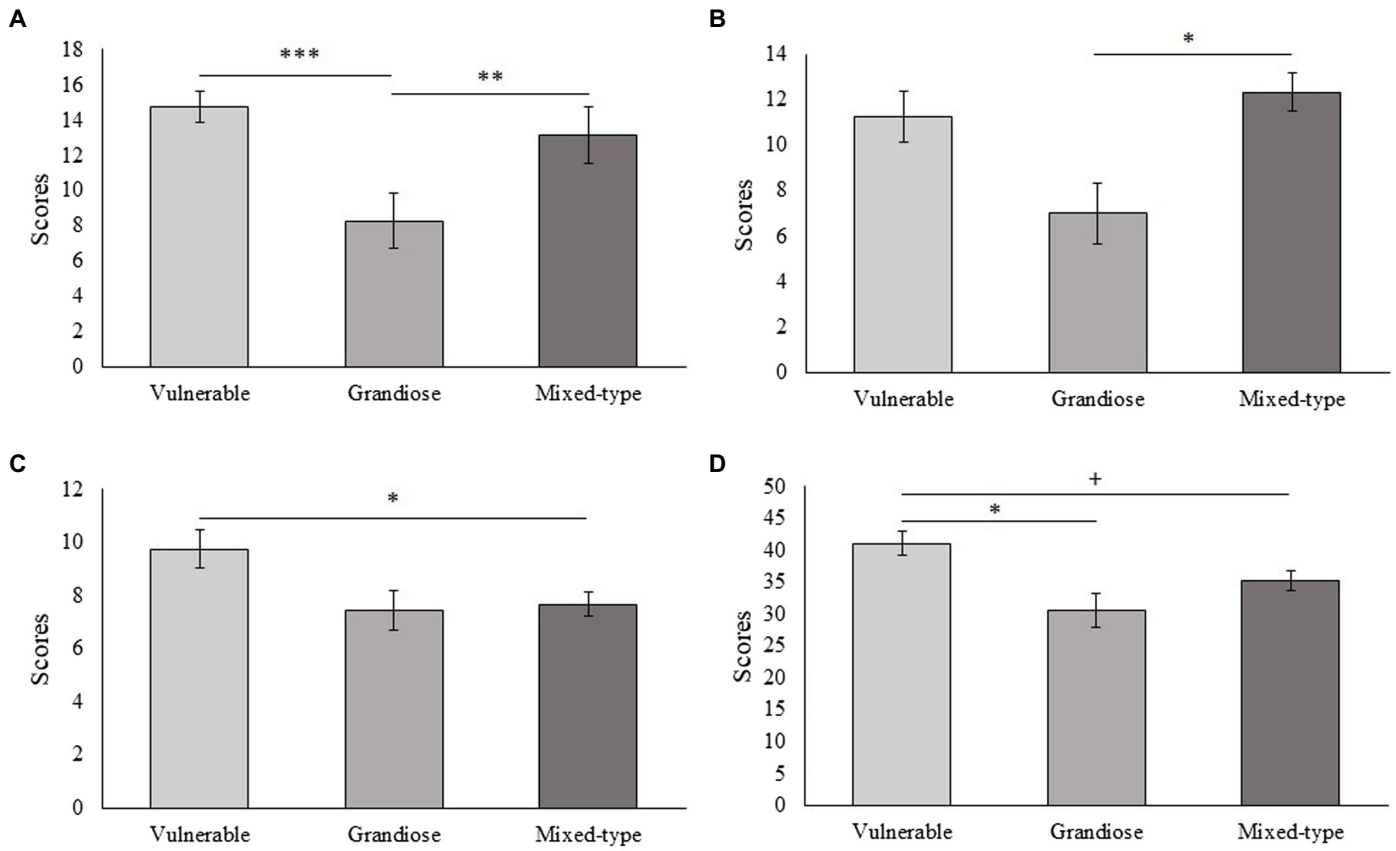
Differences between the three narcissism categories on the schizotypal traits. **(A)** Social anxiety [*F*_(2,44)_ = 9.259, *p* < 0.001, *η*^2^ = 0.296]. **(B)** Eccentric behavior [*F*_(2,44)_ = 4.482, *p* = 0.017, *η*^2^ = 0.169]. **(C)** Constricted affect [*F*_(2,44)_ = 3.598, *p* = 0.036, *η*^2^ = 0.141]. **(D)** Interpersonal [*F*_(2,44)_ = 4.657, *p* = 0.015, *η*^2^ = 0.175]. *Bonferroni *p* < 0.05, **Bonferroni *p* < 0.01, ***Bonferroni *p* < 0.001, +Bonferroni *p* < 0.09.

**Figure 4:**
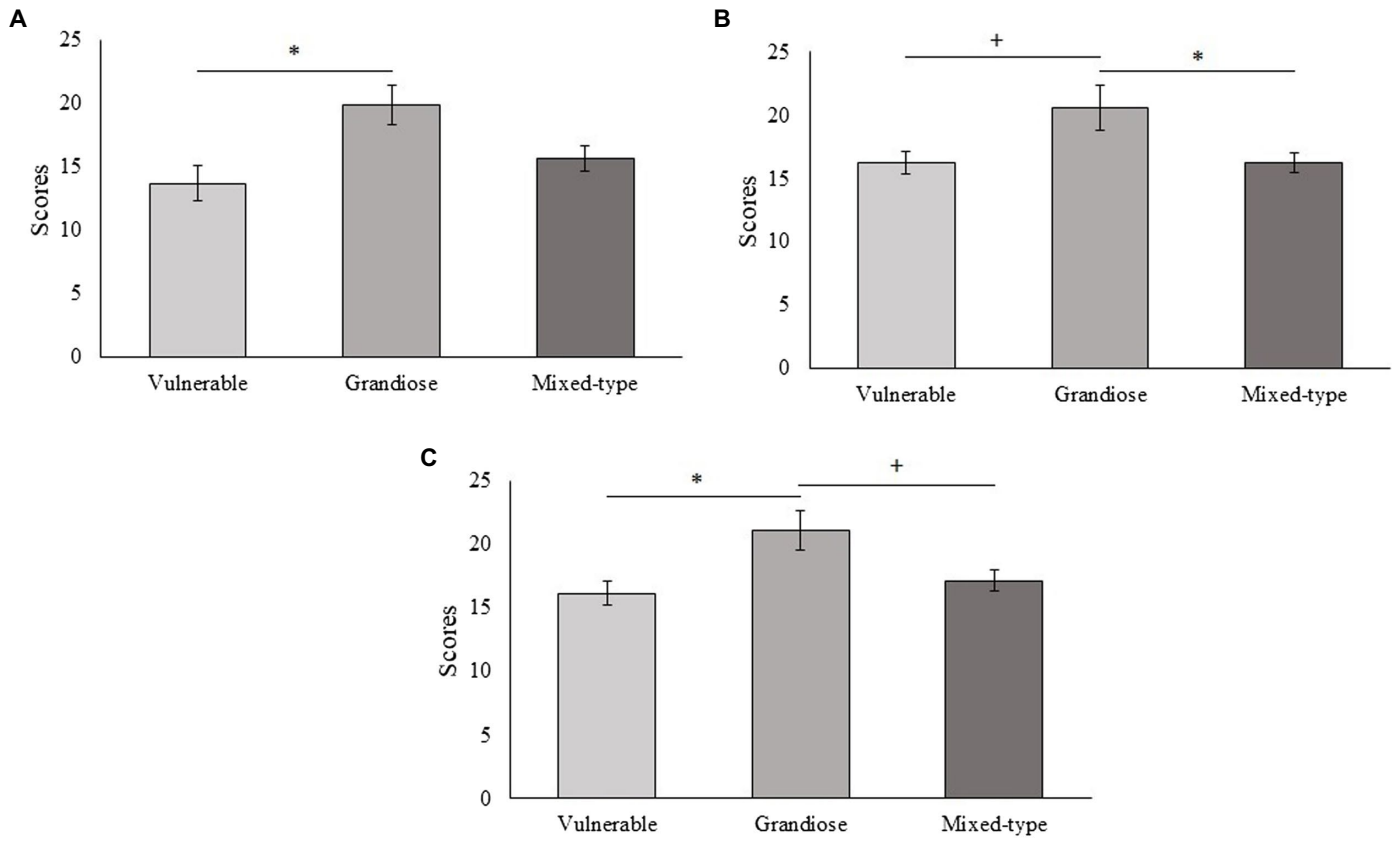
Differences between the three narcissism categories on the psychopathic traits. **(A)** Fearlessness [*F*_(2,44)_ = 3.499, *p* = 0.039, *η*^2^ = 0.137]. **(B)** Stress immunity [*F*_(2,44)_ = 3.518, *p* = 0.038, *η*^2^ = 0.138]. **(C)** Social influence [*F*_(2,44)_ = 3.488, *p* = 0.039, *η*^2^ = 0.137]. *Bonferroni *p* < 0.05, +Bonferroni *p* < 0.09.

Subjects with vulnerable narcissistic traits scored higher in the schizotypal traits “social anxiety” (*p* < 0.001) and “interpersonal” (*p* = 0.017) and tended to score lower in extraversion (*p* = 0.060) than subjects with grandiose narcissistic traits. Furthermore, subjects with vulnerable narcissistic traits scored higher in conscientiousness (*p* = 0.035) and in the schizotypal trait “constricted affect” (*p* = 0.045) and tend to score higher in “interpersonal” (*p* = 0.087) than subjects with a mixed-type of narcissism (Figure 3). Subjects with grandiose narcissistic traits scored higher than subjects with vulnerable narcissistic traits in the psychopathic traits “fearlessness” (*p* = 0.035) and “social influence” (*p* = 0.043). There was also a tendency for subjects high in grandiose narcissism to score higher than subjects high in vulnerable narcissism in the psychopathic trait “stress immunity” (*p* = 0.079; Figure 4). Medium-to-large effects were found for all these associations (Table 2).

**Table 2:** Descriptive statistics for ANOVAs between the subtypes of narcissism.

	Vulnerable *M* (*SD*)	Grandiose *M* (*SD*)	Mixed-type *M* (*SD*)	*F*_(*2*,*44*)_, *p*, *η*^2^
SPQ ideas of reference	8.17 (2.75)	7.00 (3.46)	8.21 (3.04)	4.299, 0.631, 0.021
SPQ social anxiety	14.75 (2.98)	8.29 (4.07)	13.14 (3.08)	9.259, < 0.001, 0.296
SPQ odd beliefs	6.83 (2.88)	7.71 (3.27)	7.50 (4.22)	0.159, 0.853, 0.007
SPQ unusual experiences	10.17 (3.71)	8.29 (2.43)	9.75 (4.00)	0.585, 0.561, 0.026
SPQ eccentric behavior	11.25 (3.84)	7.00 (3.55)	12.32 (4.47)	4.482, 0.017, 0.169
SPQ no close friends	8.42 (2.71)	5.71 (2.69)	6.54 (3.00)	2.465, 0.097, 0.101
SPQ odd speech	7.08 (2.81)	5.86 (1.77)	7.61 (2.62)	1.310, 0.280, 0.056
SPQ constricted affect	9.75 (2.49)	7.43 (1.98)	7.68 (2.40)	3.598, 0.036, 0.141
SPQ paranoid ideation	8.17 (1.89)	9.14 (2.85)	7.82 (2.72)	0.750, 0.478, 0.033
SPQ cognitive perceptual	33.33 (7.54)	32.14 (9.72)	33.29 (12.43)	0.033, 0.968, 0.001
SPQ interpersonal	41.08 (6.40)	30.57 (7.25)	35.18 (8.07)	4.657, 0.015, 0.175
SPQ disorganized	13.92 (5.07)	13.57 (3.95)	15.11 (6.27)	0.308, 0.736, 0.014
SPQ score	88.00 (14.51)	68.43 (16.14)	83.21 (22.22)	2.248, 0.118, 0.093
PPI Machiavellian egocentricity	14.50 (3.89)	16.29 (2.69)	14.86 (4.06)	0.507, 0.606, 0.023
PPI fearlessness	13.67 (4.79)	19.86 (4.01)	15.64 (5.17)	3.499, 0.039, 0.137
PPI rebellious non-conformity	12.83 (3.66)	15.14 (2.19)	14.93 (5.19)	0.998, 0.377, 0.043
PPI blame externalization	13.00 (3.43)	12.57 (5.22)	13.07 (5.45)	0.028, 0.972, 0.001
PPI stress immunity	16.25 (2.92)	20.57 (4.68)	16.29 (4.13)	3.518, 0.038, 0.138
PPI cold heartedness	15.08 (4.46)	13.29 (5.46)	13.93 (3.55)	0.506, 0.606, 0.022
PPI social influence	16.17 (3.21)	21.14 (4.10)	17.18 (4.40)	3.488, 0.039, 0.137
PPI carefree non-planfulness	11.58 (2.77)	11.00 (4.16)	11.46 (3.00)	0.082, 0.921, 0.004
PPI general score	113.08 (13.23)	129.86 (12.61)	117.36 (17.17)	2.607, 0.085, 0.106
STAI	20.00 (2.21)	53.14 (1.46)	50.29 (3.75)	2.576, 0.088, 0.105
AQ	23.25 (4.30)	19.14 (4.45)	20.79 (5.05)	1.841, 0.171, 0.077
Honesty-Humility	32.67 (7.22)	27.57 (7.04)	34.21 (6.54)	2.687, 0.079, 0.109
Emotionality	34.67 (4.11)	28.57 (4.07)	34.07 (6.65)	2.899, 0.066, 0.116
Extraversion	32.17 (4.52)	38.14 (6.12)	32.82 (5.25)	3.407, 0.042, 0.134
Agreeableness	31.42 (4.88)	31.29 (7.71)	32.32 (5.63)	0.154, 0.857, 0.007
Conscientiousness	39.75 (5.17)	38.00 (4.20)	34.71 (5.94)	3.766, 0.031, 0.146
Openness to experience	38.75 (4.69)	36.43 (9.72)	36.61 (5.72)	0.553, 0.579, 0.025

M, mean; SD, standard deviation; SPQ, schizotypal personality questionnaire; PPI, psychopathic personality inventory; STAI, trait anxiety inventory; AQ, autism spectrum quotient.

Subjects with a mixed-type of narcissism scored higher than subjects with grandiose narcissistic traits in the schizotypal traits “social anxiety” (*p* = 0.003), “eccentric behavior” (*p* = 0.014; Figure 3), and lower than subjects with grandiose narcissistic traits in the psychopathic trait “stress immunity” (*p* = 0.041; Figure 4). Medium-to-large effects were found for all these associations (Table 2). No significant results were found for the STAI, AQ, and the other variables of SPQ, PPI, and HEXACO (*p* > 0.05 in all cases). It is worth highlighting that effect sizes for the schizotypal trait “no close friends,” the general psychopathy score, and the STAI were also medium-to-large despite not having reached statistical significance (Table 2), which could be informative about further differences between narcissism subtypes.

### 3.4. Changes in emotions^3^

Participants reported a significant decrease in happiness scores (*t* = 3.296, *p* = 0.002, *d* = 0.48) and significant increases in scores of anxiety (*t* = −7.269, *p* < 0.001, *d* = 1.06), anger (*t* = −2.595, *p* = 0.013, *d* = 0.40), shame (*t* = −4.245, *p* < 0.001, *d* = 0.62), shame behavior (*t* = −5.422, *p* < 0.001, *d* = 0.79), and devaluation (*t* = −6.448, *p* < 0.001, *d* = 0.94) after the TSST. No significant differences for sadness were found (*t* = −0.443, *p* = 0.660). Broken out by subtype of narcissism, we found that subjects with vulnerable narcissistic traits experienced significant increases in anxiety (*t* = −3.156, *p* = 0.009, *d* = 0.91), anger (*t* = −2.449, *p* = 0.032, *d* = 0.70), shame behavior (*t* = −2.729, *p* = 0.020, *d* = 0.78), and devaluation (*t* = −2.291, *p* = 0.043, *d* = 0.66), whereas subjects with grandiose narcissistic traits only experienced a significant increase in devaluation (*t* = −3.074, *p* = 0.022, *d* = 1.16) and a tendency to increase anxiety (*t* = −2.183, *p* = 0.072, *d* = 0.82). Similar to the observed results for the whole sample, subjects with a mixed-type of narcissism experienced a decrease in happiness (*t* = 3.256, *p* = 0.003, *d* = 0.61) and increases in anxiety (*t* = −6.806, *p* < 0.001, *d* = 1.28), shame (*t* = −3.621, *p* = 0.001, *d* = 0.68), shame behavior (*t* = −4.457, *p* < 0.001, *d* = 0.84), and devaluation (*t* = −5.533, *p* < 0.001, *d* = 1.04).

## 4. Discussion

Consistent with our hypothesis of divergent mechanisms of psychobiological stress-coping in grandiose and vulnerable narcissism, the present exploratory study found a strong increase in cortisol levels after the TSST in subjects high in vulnerable narcissism, whereas a blunted response was observed in subjects high in grandiose narcissism. This significant difference in cortisol responses between subjects with vulnerable and grandiose narcissistic traits was consistent with our first prediction and emerged from the data with medium-to-large effect sizes despite the small sample size. One of the strengths of the present study, is that our results show that the observed difference in cortisol reactivity was not a byproduct of differences in extraversion/introversion, because the difference remained statistically significant even after controlling for this variable in the analysis. In support of our second prediction, we found that subjects with vulnerable narcissistic traits showed higher scores on the “social anxiety” and “interpersonal” dimensions of schizotypy, and reported increases in negative emotions such as anxiety, anger, shame behavior, and devaluation after the TSST, whereas subjects with grandiose narcissistic traits showed higher scores on the “fearlessness” and “social influence” dimensions of psychopathy and reported an increase in devaluation scores after the TSST, but not in anxiety. These findings provide additional validation for the two subtypes of narcissism. Additionally, our results also point to the occurrence of a third subtype of narcissism, the mixed-type, which is characterized by joint traits of both vulnerable and grandiose narcissism (see also Campbell and Crist, 2020). This conclusion is supported by our findings showing that the mixed-type narcissism can be physiologically and psychologically differentiated from the other two subtypes in some traits. For example, subjects in the mixed-type group scored higher in the schizotypal traits of “social anxiety” and “eccentric behavior” and lower in the psychopathic trait “stress immunity” than subjects high in grandiose narcissism, and that the mixed-type tended to have a smaller cortisol response than subjects high in vulnerable narcissism. Additionally, the medium-to-large effect sizes found in non-significant results may be indicative of further psychological and personality differences between the narcissism subtypes.

One of the main characteristics of subjects high in vulnerable narcissism is a high sensitivity to social criticism and concomitant fear to be exposed (Twenge and Campbell, 2003; Miller et al., 2011). Subjects high in vulnerable narcissism may show stronger cortisol responses to the TSST because their unstable self-image is generally associated with anxiety and fear of challenges. The observed high scores of social anxiety and constricted affect as well as the increases in negative emotions within this subtype of narcissism are consistent with this explanation. In contrast, inflated ego, exhibitionism, and vanity are common characteristics of subjects high in grandiose narcissism (Jones and Paulhus, 2011). Subjects with grandiose narcissistic traits may not react with anxiety and fear to social evaluation because, similar to psychopaths, they are relatively fearless in socially challenging situations. Thus, it is possible that the performance in front of an audience that is part of the TSST procedure is not interpreted by them as anxiety-provoking or stressful, but it may even reinforce their vanity and competitiveness (see Wallace and Baumeister, 2002). The observed higher scores of social influence, fearlessness, and stress immunity, as well as the lack of increases in the vast majority of negative emotions in the subjects high in grandiose narcissism, may have played a role in their observed blunted cortisol response to the TSST. Consistent with these explanations, we found a weak cortisol response to the TSST in the group of subjects with a mixed-type of narcissism, who shared some psychological traits (e.g., in terms of schizotypy, psychopathy, and negative emotions) with subjects within the vulnerable and the grandiose subtypes of narcissism (see Campbell and Crist, 2020, for a discussion of subjects who show both grandiose and vulnerable narcissistic traits).

Previous research has reported that subjects high in grandiose narcissism tend to show positive affect after stress or following provocation (Wolven, 2015; Hart et al., 2021), are more resilient and have higher adaptive capacity (Sękowski et al., 2021), and are psychologically healthier and report lower perceived stress and higher life satisfaction (Ng et al., 2014) than subjects high in vulnerable narcissism. However, previous research on the association between cortisol reactivity to stress and narcissism has not accounted for narcissism subtypes.

Our findings may help to explain why the results of some previous studies are inconsistent. For example, two studies found that individuals with grandiose narcissism exhibited elevated cortisol response after the TSST (Edelstein et al., 2010; Cheng et al., 2013) while a third found that cortisol levels decreased after a social evaluative test (lying while being videotaped; Dane et al., 2018). Our results suggest that future studies examining the role of stress-reactivity in NPD and non-pathological narcissism, should take the vulnerable subtype, and even the mixed-type into consideration. The results could be seen as supporting some previous reports of elevated interpersonal hypersensitivity in NPD, such as the finding of increased oxidative stress in NPD and borderline personality disorder, but not other personality disorders (Lee et al., 2020). On the other hand, the results may also point to clear biological boundaries between vulnerable and grandiose narcissism, and potentially less clear boundaries in the mixed-type, with grandiose narcissism lacking interpersonal hypersensitivity with blunted cortisol responses under socially stressful situations. Future studies of narcissism should continue to investigate possible differences in the (physiological, and perhaps also emotional-cognitive) mechanisms underlying grandiose, vulnerable, and the mixed-type narcissism as well as possible differences in their adaptive significance: for example, the extent to which they are accompanied by similar or different sociosexual strategies (e.g., Jonason et al., 2009) that modulate mate value and number of sexual partners (Borráz-León and Rantala, 2021; Burtăverde et al., 2021) in subjects with these narcissism subtypes.

### 4.1. Limitations

The use of college students may be a limitation since the high homogeneity of the sample limits generalizability. The small sample size is another important limiting factor in this study. However, statistically significant results have also been observed in similar studies with small sample sizes (e.g., Young and Nolen-Hoeksema, 2001; Dane et al., 2018; Walter et al., 2018; Zhang et al., 2019). In any case, further studies with larger and more heterogeneous samples are needed to support or reject the hypotheses raised in this study as well as to increase the generalizability of the results to other populations. Although no sex differences in cortisol response or narcissism subtypes were found in the present study, the role of sex in the association between cortisol reactivity and narcissism should be further investigated, as previous studies have reported differences in narcissism scores between men and women (Borráz-León et al., 2019; see Muris et al., 2017 for a meta-analysis) whereas others have not found these differences (Borráz-León and Rantala, 2021; Burtăverde et al., 2021). The use of stress-inducing procedures other than the TSST, would also be welcome, since different cortisol changes have been observed in response to different stressful procedures (Coleman et al., 2019). Longitudinal studies in which cortisol and personality traits are measured several times over long periods of time are also needed to explore, for example, changes in cortisol reactivity from childhood to adolescence, since previous research has reported blunted or decreased cortisol responses in subjects with chronic stress (e.g., Borráz-León et al., 2017; Lam et al., 2019), which might also explain the blunted cortisol reactivity in grandiose narcissists as a coping mechanism in stressful situations. Finally, since this is an exploratory study with a small sample size, the results must be interpreted with caution. Nevertheless, the results will be informative for future confirmatory research conducted with larger and more heterogeneous samples using preregistration of hypotheses and predictions derived from theory and from the results of this exploratory study.

### 4.2. Conclusion

The results of our study show that whereas subjects with vulnerable narcissistic traits had strong cortisol and emotional reactivity to psychosocial stress and psychological characteristics associated with schizotypy, subjects with grandiose narcissistic traits had blunted hormonal and emotional reactivity to psychosocial stress and psychological characteristics associated with psychopathy. The mixed-type arose as a potential third narcissism subtype with subjects characterized by showing a mixture of both vulnerable and grandiose narcissistic traits. This exploratory study provides the first evidence of differential physiological and emotional reactivity to social evaluation threat in subjects with vulnerable and grandiose narcissism and may enhance our understanding of narcissism and its multiple expressions.

## Data availability statement

The original contributions presented in the study are included in the article/Supplementary material, further inquiries can be directed to the corresponding author.

## Ethics statement

The studies involving human participants were reviewed and approved by the Social Science Institutional Review Board at the University of Chicago. The patients/participants provided their written informed consent to participate in this study.

## Author contributions

JIB-L: formal analysis, investigation, writing—original draft, and writing—review and editing. AS: formal analysis and writing—review and editing. CS and ML: conceptualization, investigation, methodology, and writing—review and editing. RL: writing—original draft and writing—review and editing. DM: conceptualization, investigation, resources, writing—original draft, and writing—review and editing. All authors contributed to the article and approved the submitted version.

## Conflict of interest

The authors declare that the research was conducted in the absence of any commercial or financial relationships that could be construed as a potential conflict of interest.

## Publisher’s note

All claims expressed in this article are solely those of the authors and do not necessarily represent those of their affiliated organizations, or those of the publisher, the editors and the reviewers. Any product that may be evaluated in this article, or claim that may be made by its manufacturer, is not guaranteed or endorsed by the publisher.

## Footnotes

^1^Similar values of vulnerable narcissism have been reported in previous studies (e.g., Zajenkowski and Szymaniak, 2021: *M* = 30.49, *SD* = 6.35; *t* = −0.569, *p* = 0.569 vs. our sample).

^2^Similar values of grandiose narcissism have been reported in previous studies (e.g., Cheng et al., 2013: *M* = 14.30, *SD* = 7.29; *t* = −0.090, *p* = 0.920 vs. our sample).

^3^A reviewer requested us to analyze correlations between cortisol reactivity and emotional reactivity between the subtypes of narcissism. No significant associations were found for any of the narcissism subtypes (*r* < 0.1, *p* > 1.0 in all cases).
