# Supplementary material for: Cortisol reactivity to psychosocial stress in vulnerable and grandiose narcissists: An exploratory study
Source: Front Psychol. 2023 Jan 6;13:1067456. doi: 10.3389/fpsyg.2022.1067456 (PMC9852882; doi:10.3389/fpsyg.2022.1067456)
Supplement: Supplementary file 2 [file Table_1.DOCX]

Supplementary Table 1. Sex differences in the study variables.

| **Variables** | **Mean (*SD*)** | | ***t*** | ***p*** |  |
| --- | --- | --- | --- | --- | --- |
|  | **Men (*n* = 17)** | **Women  (*n* = 30)** |  |  | **Cohen’s *d*** |
| **Pre-TSST cortisol** | -0.732 (0.35) | -0.858 (0.39) | 1.117 | 0.271 | 0.34 |
| **Post-TSST cortisol** | -0.584 (0.51) | -0.765 (0.35) | 1.282 | 0.212 | 0.41 |
| **Cortisol change** | 0.150 (0.29) | 0.096 (0.20) | 0.670 | 0.509 | 0.21 |
| **HSNS** | 29.12 (5.75) | 30.40 (5.88) | -0.728 | 0.472 | 0.22 |
| **NPI** | 14.00 (6.89) | 14.67 (6.68) | -0.322 | 0.749 | 0.09 |
| **SPQ ideas of reference** | 7.76 (3.25) | 8.17 (2.90) | -0.423 | 0.675 | 0.13 |
| **SPQ social anxiety** | 12.82 (4.14) | 12.83 (3.58) | -0.008 | 0.994 | 0.00 |
| **SPQ odd beliefs** | 6.24 (3.07) | 8.00 (4.05) | -1.681 | 0.100 | 0.48 |
| **SPQ unusual experiences** | 9.12 (3.68) | 9.93 (3.76) | -0.723 | 0.475 | 0.21 |
| **SPQ eccentric behavior** | 12.29 (4.48) | 10.67 (4.49) | 1.194 | 0.241 | 0.36 |
| **SPQ no close friends** | 7.18 (2.72) | 6.73 (3.15) | 0.506 | 0.616 | 0.15 |
| **SPQ odd speech** | 6.29 (2.20) | 7.73 (2.69) | -1.984 | 0.054 | 0.58 |
| **SPQ constricted affect** | 8.65 (2.23) | 7.90 (2.64) | 1.029 | 0.310 | 0.30 |
| **SPQ paranoid ideation** | 7.41 (2.47) | 8.50 (2.54) | -1.434 | 0.161 | 0.43 |
| **SPQ cognitive perceptual** | 30.53 (9.59) | 34.60 (11.32) | -1.307 | 0.199 | 0.38 |
| **SPQ interpersonal** | 36.06 (7.28) | 35.97 (8.73) | 0.039 | 0.969 | 0.01 |
| **SPQ disorganized** | 12.53 (4.51) | 15.73 (5.93) | -2.079 | **0.044** | 0.60 |
| **SPQ score** | 80.47 (18.18) | 83.23 (21.66) | -0.466 | 0.644 | 0.13 |
| **PPI Machiavellian egocentricity** | 14.59 (2.91) | 15.20 (4.97) | -0.581 | 0.565 | 0.14 |
| **PPI fearlessness** | 16.24 (5.40) | 15.50 (5.15) | 0.456 | 0.652 | 0.14 |
| **PPI rebellious nonconformity** | 14.71 (4.95) | 14.27 (4.36) | 0.304 | 0.763 | 0.09 |
| **PPI blame externalization** | 12.35 (4.94) | 13.33 (4.90) | -0.655 | 0.517 | 0.19 |
| **PPI stress immunity** | 18.71 (3.07) | 15.90 (4.39) | 2.560 | **0.014** | 0.74 |
| **PPI cold heartedness** | 14.94 (4.84) | 13.67 (4.54) | 0.951 | 0.351 | 0.27 |
| **PPI social influence** | 17.00 (4.70) | 17.80 (4.13) | -0.585 | 0.563 | 0.18 |
| **PPI carefree nonplanfulness** | 10.29 (2.49) | 12.07 (3.21) | -2.103 | **0.042** | 0.61 |
| **PPI general score** | 118.82 (14.16) | 117.73 (17.52) | 0.232 | 0.818 | 0.06 |
| **STAI** | 50.76 (2.81) | 50.57 (3.57) | 0.209 | 0.835 | 0.05 |
| **AQ** | 20.41 (4.19) | 21.60 (5.25) | -0.849 | 0.401 | 0.25 |
| **Honesty-Humility** | 35.82 (4.29) | 31.13 (7.74) | 2.671 | **0.010** | 0.74 |
| **Emotionality** | 29.59 (4.71) | 35.57 (5.66) | -3.875 | **<0.001** | 1.14 |
| **Extraversion** | 32.65 (5.11) | 33.90 (5.70) | -0.774 | 0.444 | 0.23 |
| **Agreeableness** | 32.94 (4.98) | 31.37 (6.05) | 0.962 | 0.342 | 0.28 |
| **Conscientiousness** | 36.94 (6.46) | 36.23 (5.60) | 0.378 | 0.708 | 0.11 |
| **Openness to experience** | 39.18 (5.96) | 35.97 (6.03) | 1.766 | 0.087 | 0.53 |
| **Age** | 27.18 (9.11) | 22.63 (3.46) | 1.975 | 0.063 | 0.66 |
