# Supplementary material for: Cortisol reactivity to psychosocial stress in vulnerable and grandiose narcissists: An exploratory study
Source: Front Psychol. 2023 Jan 6;13:1067456. doi: 10.3389/fpsyg.2022.1067456 (PMC9852882; doi:10.3389/fpsyg.2022.1067456)
Supplement: Supplementary file 3 [file Table_2.DOCX]

Supplementary Table 2. Partial correlations controlling for age and sex between cortisol levels (pre-TSST, post-TSST, and change) and psychological variables.

| **Variables** | **Pre-TSST cortisol *r* (*p*)** | **Post-TSST cortisol *r* (*p*)** | **Cortisol change  *r* (*p*)** |
| --- | --- | --- | --- |
| **HSNS** | -0.151 (0.321) | 0.022 (0.887) | 0.287 (0.056) |
| **NPI** | 0.071 (0.643) | -0.137 (0.369) | **-0.353 (0.017)** |
| **SPQ ideas of reference** | **-0.294 (0.050)** | -0.257 (0.089) | 0.027 (0.863) |
| **SPQ social anxiety** | **-0.351 (0.018)** | -0.081 (0.597) | **0.427 (0.003)** |
| **SPQ odd beliefs** | 0.019 (0.902) | 0.017 (0.912) | 0.001 (0.995) |
| **SPQ unusual experiences** | 0.027 (0.860) | 0.075 (0.623) | 0.089 (0.559) |
| **SPQ eccentric behavior** | -0.144 (0.346) | -0.154 (0.311) | -0.039 (0.798) |
| **SPQ no close friends** | -0.026 (0.865) | 0.053 (0.732) | 0.138 (0.367) |
| **SPQ odd speech** | -0.061 (0.689) | 0.111 (0.467) | **0.295 (0.049)** |
| **SPQ constricted affect** | 0.020 (0.897) | 0.062 (0.687) | 0.076 (0.618) |
| **SPQ paranoid ideation** | 0.096 (0.529) | 0.100 (0.515) | 0.021 (0.892) |
| **SPQ cognitive perceptual** | -0.044 (0.773) | -0.017 (0.911) | 0.043 (0.778) |
| **SPQ interpersonal** | -0.135 (0.377) | 0.032 (0.836) | 0.276 (0.066) |
| **SPQ disorganized** | -0.016 (0.919) | 0.063 (0.681) | 0.137 (0.368) |
| **SPQ score** | -0.137 (0.369) | -0.035 (0.819) | 0.162 (0.287) |
| **PPI Machiavellian egocentricity** | -0.090 (0.556) | -0.136 (0.375) | -0.087 (0.568) |
| **PPI fearlessness** | -0.038 (0.803) | -0.200 (0.187) | -0.286 (0.057) |
| **PPI rebellious nonconformity** | -0.092 (0.549) | -0.205 (0.176) | -0.210 (0.165) |
| **PPI blame externalization** | 0.209 (0.168) | 0.208 (0.170) | 0.028 (0.857) |
| **PPI stress immunity** | **0.303 (0.043)** | 0.247 (0.102) | -0.053 (0.730) |
| **PPI cold heartedness** | 0.032 (0.836) | 0.078 (0.611) | 0.091 (0.554) |
| **PPI social influence** | 0.110 (0.471) | 0.005 (0.975) | -0.168 (0.271) |
| **PPI carefree nonplanfulness** | -0.054 (0.724) | 0.113 (0.459) | 0.289 (0.054) |
| **PPI general score** | 0.103 (0.502) | 0.009 (0.953) | -0.144 (0.346) |
| **STAI** | -0.180 (0.236) | -0.200 (0.187) | -0.060 (0.697) |
| **AQ** | -0.122 (0.426) | -0.014 (0.929) | 0.169 (0.267) |
| **Honesty-Humility** | 0.097 (0.526) | 0.182 (0.230) | 0.162 (0.288) |
| **Emotionality** | 0.074 (0.628) | 0.144 (0.344) | 0.128 (0.404) |
| **Extraversion** | 0.103 (0.501) | -0.012 (0.938) | -0.187 (0.220) |
| **Agreeableness** | 0.016 (0.916) | 0.005 (0.975) | -0.017 (0.914) |
| **Conscientiousness** | 0.172 (0.259) | 0.194 (0.201) | 0.060 (0.696) |
| **Openness to experience** | -0.001 (0.994) | 0.016 (0.914) | 0.029 (0.852) |
